# Delocalized Plastic Flow in Proton-Irradiated Monolithic Metallic Glasses

**DOI:** 10.1038/srep23244

**Published:** 2016-03-18

**Authors:** Jaewon Heo, Sunghwan Kim, Seunghwa Ryu, Dongchan Jang

**Affiliations:** 1 Department of Nuclear and Quantum Engineering, Korea Advanced Institute of Science and Technology, Daejeon, 34141, South Korea; 2 Department of Mechanical Engineering, Korea Advanced Institute of Science and Technology, Daejeon, 34141, South Korea

## Abstract

Creating new materials with novel properties through structural modification is the Holy Grail of materials science. The range of targetable structures for amplification of mechanical properties in metallic glasses would include types of atomic short range orders at the smallest scale through compositions or morphologies of phases in composites. Even though the usefulness of the latter approach has been successfully demonstrated in the past decades, the feasibility of the former has been incompletely proved with only marginal property improvements reported within experimentally-accessible atomic-level structural changes. Here, we report the significant enhancement of deformability in Zr-based monolithic metallic glass only through the atomic disordering by proton irradiation without altering any other structural traits. Metallic glass nanopillars that originally failed catastrophically without any notable plasticity become capable of attaining more than 30% uniaxial plastic strain accommodated by homogeneous deformation when irradiated to ~1 displacement per atom (DPA). We discuss the atomistic origin of this improved plasticity in terms of density and spatial distributions of icosahedral short range order influenced by irradiation.

The basics of materials science involve investigation of materials’ properties in relation to their structures^1^. For example, mechanical properties of conventional crystalline metals, such as strength and ductility, are closely dependent on the local deficiency of crystallographic symmetries, such as dislocations or vacancies, whose spatial arrangement prescribes a structure of materials. On the contrary, metallic glasses, due to the absence of the long-range translational order, have clearly distinguished structural features from those of their crystalline counterparts, wherein the heterogeneity in local atomic configuration associated with the fluctuation of short- and medium-range orders^2^ appears to play the central role^3^,^4^. Widely-used structural parameters that dictate such atomic-level structures in metallic glasses include average value and distribution of the free volume^3^,^5^ or the type and fraction of the coordination polyhedra formed with atoms at the vertex positions^2^,^6^. Then, the relationship between mechanical properties and structures of metallic glasses can be established by scrutinizing how such structural parameters are correlated with atomic-level straining processes under a given stress condition. Local clusters of ~100 atoms susceptible to cooperative rearrangements by the applied stress serve as basic plasticity units for metallic glasses, called shear transformation zones (STZs)^3^,^4^, which preferentially occur in compromised regions of low activation barriers characterized by large excessive free volumes^3^,^4^ or high disorderedness with broken local symmetries^7^,^8^,^9^. Dynamic aspects including formation, destruction, and mutual interaction of many STZs that link microscopic shearing events to the macroscopic deformation mode constitute the primordial part in understanding mechanical properties of metallic glasses. At room temperature, for example, STZs generally tend to cohere within a narrow area resulting in the rapid shear localization by shear banding^10^. In many cases, this shear banding phenomenon is coupled with ceramic-like catastrophic failure and this is a serious downside of bulk metallic glasses for structural applications.

Analogous to crystalline metals in which the change of defect structures by thermomechanical treatments has been proved to be a useful technique to control the mechanical behavior^1^, manipulation of atomic-level structure can also offer an opportunity to tune the plastic deformation of metallic glasses. For example, structural or chemical disordering of metallic glasses by mechanical work^11^,^12^, high energy particle irradiation^13^,^14^,^15^,^16^,^17^, or extremely fast vitrification^7^,^8^,^18^ can cause the change in strength and deformability. On the other hand, structural relaxation by thermal^12^,^19^ or mechanical^20^ annealing reverses this change. Given that the brittle nature of metallic glasses typically arises from rapid shear localization, alteration of atomic-level structures to obtain a new glassy state capable of delocalizing plastic flow could give us a promise to overcome the issue of catastrophic failure occurring in many metallic glasses. In this study, we irradiated a Zr_57_Nb_5_Al_10_Cu_15.4_Ni_12.6_ metallic glass sample with protons of 6 discrete energies between 30 and 200 keV to ~1 DPA damage level (Fig. 1f) and investigated the change in the strength and deformability by performing nano-compression experiments on the cylindrical nanopillars with 200 and 400 nm diameters. We demonstrated that in sharp contrast to the non-existence of macroscopic plasticity in as-fabricated metallic glass samples, the irradiated ones can attain drastically increased homogeneous deformation up to ~30% plastic strain, extraordinarily high value for a monolithic metallic glass. Experimental results were then further analyzed by molecular dynamics (MD) simulations.

Figure 1a shows the schematic illustration for the experimental setup of proton irradiation and nanopillar fabrication. The bulk metallic glass specimen was sequentially irradiated using 6 different proton energies so as for the superimposed radiation damage profile from each proton energy to be nearly uniform down to ~1.4 *μ*m from the surface (Fig. 1f). Figure 1b shows the cross-sectional scanning electron microscope (SEM) image. The electron diffraction patterns in Fig. 1c,d were taken using the transmission electron microscope (TEM) from the irradiated and unirradiated regions in the same specimen, respectively. Radial intensity profiles from each electron diffraction pattern are displayed in Fig. 1g. Even though the intensity of the primary peak reduces slightly after irradiation, their locations remain nearly unchanged. This implies that irradiation abates the average number of atoms in the nearest neighbors a little but maintains their average distance constant. Figure 1e shows the representative SEM image of the 400 nm nanopillars fabricated in the irradiated layer.

Figure 2 presents the results of the nano-compression experiments on the unirradiated (top row) and irradiated (bottom row) metallic glass nanopillars with 400 nm (left section) and 200 nm (right section) diameters. SEM images in Fig. 2a,c,e,g show the representative morphologies of deformed nanopillars after nano-compression tests. It is evident from engineering stress-strain curves in Fig. 2b,f that the dominant deformation mode of the unirradiated metallic glass nanopillars is the catastrophic failure immediately following initial elastic loading without any noticeable macroscopic plasticity, typical of most bulk metallic glasses under uniaxial loading. The formation of highly localized shear bands shown in Fig. 2a,e additionally supports this conclusion. However, engineering stress-strain curves for irradiated 400 nm nanopillars exhibit clearly distinguished signature from those of unirradiated samples, in which nonlinear plasticity with intermittent stress drops (indicated by arrows in Fig. 2d) comes into existence. This quite smooth and continuous plasticity has been observed in other Zr-based metallic glass nano-pillars with diameters near 100 nm^21^, but rarely reported in monolithic bulk metallic glasses. Final failure of 400 nm irradiated samples was by rapid shear banding as seen in Fig. 2c. The transition from brittle failure to continuous plasticity is even more pronounced in 200 nm samples. Total engineering plastic strain exceeds 30% (Fig. 2h) and the deformed samples are lack of sharp shear band (Fig. 2g). Tiny stress drops in the stress-strain curves in Fig. 2h are likely corresponding to the formation of diffuse shear bands that did not lead to catastrophic failure. The emergence of non-linear plasticity due to irradiation appears to be phenomenologically similar with the result of previous studies^16^. However, it should be noted that the full irradiation over the entire specimen using high energy proton beam in this study caused significantly larger extended plasticity than what surface-only irradiation with lower energy Ga ion beam did in ref. 16. It suggests the existence of new glassy state revealed by proton irradiation, in which plasticity would occur in a completely different manner (see the discussion below).

Figure 3 presents the results of MD simulation on irradiated and unirradiated metallic glass nanopillars. Overall structural changes due to irradiation are analyzed by the pair correlation function (Fig. 3b) and histogram of Voronoi volume (Fig. 3c). The pair correlation function shows just a slight broadening of the primary peak after irradiation while its position remains nearly unchanged, consistent with our experimental electron diffraction analysis (Fig. 1g). Similarly, the peaks in the Voronoi volume histogram become slightly shorter but wider due to irradiation. These observations suggest that irradiation-induced atomic re-distribution occurred in such a way to manage the average inter-atomic distance unaltered with only a minuscule variation in the atomic free volume distribution. In this sense, it appears that a spatially-averaged structural parameter alone, such as average free volume size, is insufficient to explain the drastically increased plasticity observed in our experiments (further discussion in the later section). Figure 3d shows the engineering stress-strain relationship from the uniaxial compression tests on 10 nm diameter samples. Analogous to our experimental results, we also observed clearly contrasted plastic behavior between irradiated and unirradiated samples. While the unirradiated sample shows the upper yield strength at around 0.5% strain followed by rapid stress drop, irradiated sample exhibits much smoother and monotonically changing flow stress. Flow stresses from both samples coincide at strains higher than ~20%. The inset in Fig. 3d shows the distribution of atomic shear strains after 10% of compressive strain and highlights the differences in deformation modes. The unirradiated nanopillar makes the localized shear band upon yielding (the region between the dashed lines). On the contrary, the irradiated sample deforms uniformly via scattered atomic shear strain over the entire sample, consistent with the experimental findings (Fig. 2). We observed similar characteristics for the 5 nm diameter sample as well (see supplementary information). The computational representation of radiation-induced homogenization of plastic strains in this study is comparable with the previous work by others^15^. It should also be noted that the difference in the post-yield plasticity between the experimental (Fig. 2b,f) and computational (Fig. 3d) compression tests on the unirradiated samples is most likely due to the different loading systems. The perfectly rigid machine stiffness as well as ideal displacement control in the MD simulation enables to capture the stress-strain signal even when the significant work-softening occurs, while the finite machine stiffness and feed-back-based displacement control in the real experimental set-up limit the precise control of displacement under the same situation^22^. Furthermore, there still remains a possibility of artifacts due to many orders of magnitude difference in temporal scales between MD simulations and real experiments. Nonetheless, the inherent plastic behavior is presumably identical for both experimental and computational tests.

According to the free volume theory, the macroscopic deformation mode of metallic glasses, either inhomogeneous or homogeneous, is governed by two competing local atomic-level events: i) the structural excitation related with strain-induced free volume creation at or near the activated STZs and ii) the structural relaxation associated with free volume annihilation due to the diffusional jumps^3^. When the former becomes dominant, i.e. the free volume creation rate is higher than that of annihilation, STZs tend to agglomerate rendering macroscopic deformation to be inhomogeneous. On the other hand, when the latter prevails plastic strain diffuses out over the large volume, resulting in macroscopically homogeneous deformation^3^,^23^. Here, the structural parameter that determines the relative rates of free volume creation and annihilation is known to be γv^*^/*v*_*f*_, where *γ* is the geometric factor prescribing the shape of free volume distribution function, v^*^ is the critical volume on the order of atomic volume and *v*_*f*_ is the average free volume per atom^3^,^23^. Our experimental and computational analyses for the irradiated and unirradiated metallic glasses (Figs 1g and 3b,c) indicate that the structure of metallic glasses characterized by γv^*^/*v*_*f*_ remains nearly unchanged after the proton irradiation since there are only negligibly small changes in both the average values and distribution of free volumes. This consideration suggests that the free volume theory alone may not fully explain the origin of the significant increase of plasticity up to ~30% strain shown in Fig. 2.

Recent atomistic simulation studies discovered that types of the short-range order (SRO), characterized by the coordination polyhedrons composed of the central atom and the nearest neighbors, and their extension to form the medium-range or aperiodic long-range orders are important factors that can considerably influence the mechanical behavior of metallic glasses^2^,^7^,^8^,^18^. Especially for many Zr-based glasses, density and spatial distribution of the icosahedral short-range order are known to be the key structural features that govern the elastic rigidity^18^, strength^2^, or degree of plastic localization^8^, and therefore alteration of these parameters by thermal treatments^19^, mechanical pre-straining^11^,^12^ or irradiation^13^,^14^,^15^,^16^ can cause the different mechanical responses. Figure 4a shows the correlation of the atomic shear strains and icosahedron density distribution at different stages during compressive loading for both irradiated and unirradiated samples with 10 nm diameters (see Supplementary Information for whole movies). Obviously, irradiation decreased the initial icosahedron density as the displacement cascades due to the multiple collision events disturbed the atomic configuration and broke local symmetries. In the unirradiated sample atomic-level straining events initiate in the regions of low icosahedron density (*ε*_comp _= 5% case in Fig. 4a) and subsequently spread to the high icosahedron density zones, forming a narrow band with high shear strains (*ε*_comp_ = 10% case in Fig. 4a). During this process, atomic shear movements seem to break some icosahedral local symmetries since the regions of high shear strain and low icosahedron density coincide after plastic deformation takes place to some extent. Because it is generally known that the icosahedron configuration is energetically more stable and mechanically more robust than any other local atomic structures^2^, once a strain-induced icosahedron-depleted band traversing the entire specimen forms further plastic deformation proceeds via this easy path, keeping the flow stress low thereafter. On the contrary, because of low initial icosahedron density the irradiated sample contains a number of atomic sites capable of easy straining even prior to the mechanical testing, so that the plastic deformation can occur involving only marginal structural evolution (bottom row in Fig. 4a). Low yield strength and slowly changing flow stress in the irradiated sample are likely due to this effect.

Another important factor to consider is the spatial arrangement of icosahedral atomic units, i.e., extension of short-range orders to medium- or aperiodic long-range orders. Especially, the connectivity of interpenetrating icosahedra appears to strongly influence mechanical responses in metallic glasses^9^. The seemingly identical icosahedral unit would have higher local elastic rigidity when it belongs to the larger cluster of connected icosahedra^9^, implying that the easiness of deformation in metallic glasses may not be determined only by the number density of the icosahedral clusters but also by the way they are distributed in the specimen. According to the histogram in Fig. 4b that counts the number of agglomerated icosahedral clusters of a given size, it is evident that irradiation splits off the large icosahedral clusters into the smaller ones. Illustrations shown in Fig. 4c visually confirm this conclusion as well. This fragmentation effect could be responsible for emergence of the delocalized plastic flow in the irradiated metallic glasses. Given that the plastic yielding in metallic glasses occurs at a constant atomic shear strain, *γ*_*c*_, the activation barrier, *W*, for the local plastic shearing events in STZs linearly scales with the local elastic constant as follows^24^:


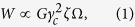


where *G* and Ω are the shear modulus and activation volume of the STZ, respectively, and ζ is a correction factor due to the confinement of the STZ in the elastic matrix. Therefore, elastic softening in the local icosahedral units due to irradiation-induced disintegration lowers the activation barrier for atomic straining in the STZ and consequently macroscopic yield strength. Because shear localization is phenomenologically related with strain-softening whose magnitude corresponds to the amount of stress drop after the yield strength (see the stress-strain curves in Fig. 3d), decrease of the yield strength approaching the subsequent flow stress attenuates the effect of strain-softening, resulting in spreading out the plastic strains over the larger volume in the specimen. However, the aforementioned discussion still remains rather phenomenological, and further study is required to elucidate the specific mechanism and energetics linking types of short range orders and their spatial and temporal distributions to mechanical responses of metallic glasses. In addition, even though this study is lack of experimental verification of delocalized flow under the tensile mode, the underlying mechanism would not be significantly influenced by the loading direction because of the symmetric nature of atomic-level shearing events with respect to the directional reversal^25^.

In summary, we demonstrate that the deformability and mechanical stability of Zr-based monolithic metallic glasses can increase drastically by engineering the atomic-level configurational structure with proton irradiation. We observe that metallic glass nanopillars undergo transition from ceramic-like brittle failure without any notable plasticity in fully annealed state to homogeneous deformation with the uniaxial engineering strain larger than 30% in disordered state by proton irradiation. We analyze the atomistic origin of this property enhancement due to the change in the icosahedral network induced by proton irradiation.

## Methods

### Experiments

**Figure 1:**
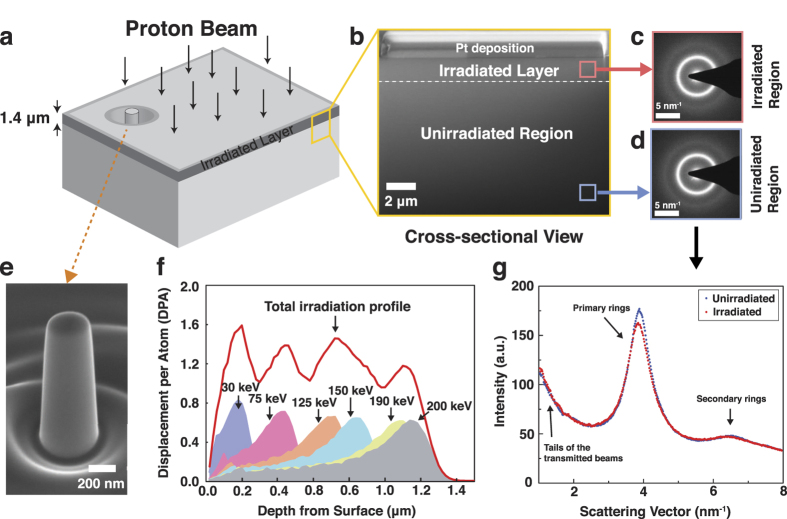
Preparation of proton-irradiated metallic glass specimen. (**a**) Schematic illustration of geometries and dimensions of irradiation process and nanopillar fabrication. (**b**) SEM image of the specimen cross-section. (**c**,**d**) Electron diffraction patterns of irradiated (**c**) and unirradiated (**d**) regions of the metallic glass specimen. (**e**) Representative SEM image of the fabricated nanopillar. (**f**) Irradiation damage profile in terms of the displacement per atom. (**g**) Intensity profile of electron diffractions calculated using the patterns in (**c**,**d**).

Two 3 mm wide and 1 mm thick square plates were cut from a Zr_57_Nb_5_Al_10_Cu_15.4_Ni_12.6_ bulk metallic glass strip. The plates were polished down to the 1 *μ*m grit coated abrasive sheet and subsequently annealed at 375 °C for 1 hour to remove any potential mechanical damages that might occur during sample preparation. One of such prepared metallic glass plates was irradiated in KAERI Advance Radiation Technology Institute with protons of 6 different energies between 30 keV and 200 keV each with a fluence of 1 × 10^17^ ions cm^−2^ (Fig. 1f). Proton irradiation profiles shown in Fig. 1f were calculated using Stoping Range of Ions in Matter (SRIM) code^26^.

Nanopillars with 200 nm and 400 nm diameters and 1:4 aspect ratio were fabricated on the surfaces of each irradiated and unirradiated plates using Helios Nanolab 450 F1 Focused Ion Beam (FIB) by sequentially applying annulus patterns with the final beam current density at 10 pA. *In situ* SEM uniaxial compression experiments were carried out using Hysitron PI 85 equipped with 2 *μ*m flat punch tip in FEI Quanta FIB. All tests were conducted at a nominally constant strain rate of 1 × 10^−3^ s^−1^ in a displacement control mode. Thermal drifts, tip and spring effects were calibrated prior to experiments to extract specimen-only responses. To ensure statistical consistency at least 10 nanopillars were tested under the same condition.

### Computations

For computational simplicity, we chose the ternary alloy system of Zr_47_Cu_46_Al_7_ composition to reasonably imitate the original Zr-based quinary metallic glass used in experiments. Large-scale Atomic/Molecular Massively Parallel Simulator (LAMMPS) was used to perform MD simulation^27^ with the embedded-atom-method (EAM) interatomic potential^28^. In addition, in order to circumvent computational artifacts that may rise due to soft cores of the EAM potential, such as an unphysical atomic overlapping, 12–6 Lennard-Jones (LJ) potential was augmented to reliably describe near-core interactions during the irradiation processes. Parameters for LJ potential (*ε* = 2.0 eV, *σ* = 1.871 Å, and *r*_*c*_ = 2.1 Å) were properly chosen so as not to interfere with the EAM potential under normal conditions^15^. Zr_47_Cu_46_Al_7_ metallic glass nanopillar was prepared by rapidly quenching from 2000K to 300K at the constant rate of 0.01K/ps while being confined in the cylindrical mold^29^. Dimensions of such prepared nanopillars are 5.1 nm (diameter) × 14.6 nm (length) and 10.1 nm (diameter) × 30.5 nm (length), possessing 17,308 and 138,608 atoms, respectively. Periodic boundary condition is applied along the nanopillar axial direction.

**Figure 3:**
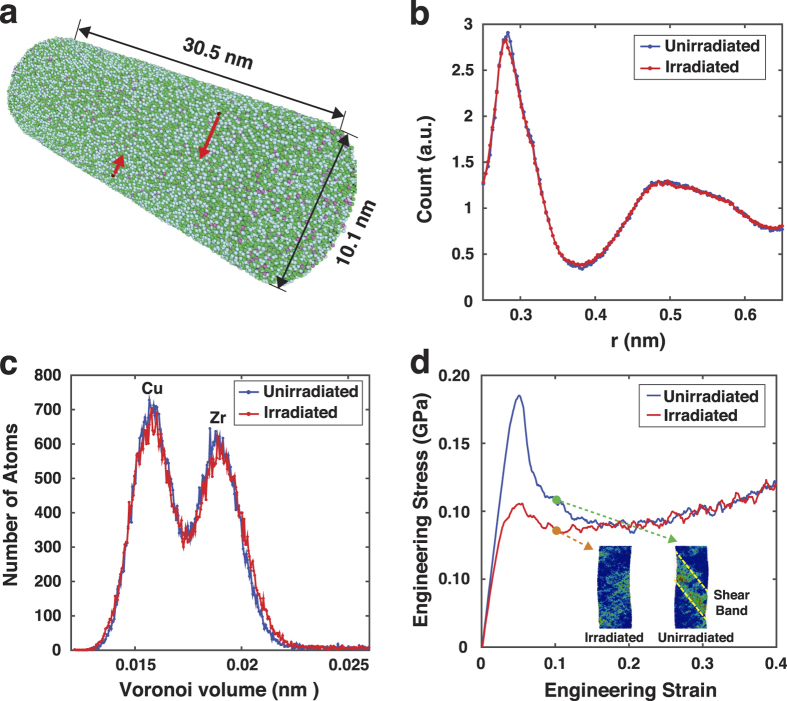
Atomistic modeling structural change of the specimen under irradiation and compressive loading. (**a**) Schematic illustration of irradiation process. (**b**) Pair correlation function and (**c**) histogram of voronoi volume of irradiated (blue) and unirradiated (red) samples. (**d**) Compressive stress-strain curves before and after irradiation. Inset represents the atomic shear strain distribution after 10% strain.

The irradiation process was modeled as a series of multiple collision events (Fig. 3a). To simulate a primary knock-on atom (PKA) comparable to the experimental irradiation process, 100 eV kinetic energy directed toward the center of nanopillar axis was added to a randomly chosen atom^13^. This process was repeated 375 and 3,000 times for the samples with 5.1 nm and 10.1 nm diameters, respectively, with 1.5 ps of adiabatic relaxation and 3.0 ps of isothermal relaxation at 300K for each iteration. Then, uniaxial compression tests were performed with constant strain rate of 1 × 10^8^ s^−1^ to 40% nominal strain. The plastic shear strain was examined by the atomic local strain tensor and the distribution of icosahedra was analyzed using cylindrical Voronoi tessellation method. Only the atoms located at the center of the icosahedral motif were counted for the icosahedral density. The atomic von Mises stress was obtained from the 6 components of virial stress of each atom, where we have used the per-atom volume obtained from the cylindrical Voronoi tessellation. Open Visualization Tool (OVITO) was used to illustrate the atomic configurations^30^. The pair correlation function shown in Fig. 3b was obtained from a separate run with all simulation conditions identical except the cylindrical specimen geometry replaced by the cube with periodic boundary condition to every dimension so as to properly describe the long-range correlation to the distance comparable to experiments.

## Additional Information

**How to cite this article**: Heo, J. *et al.* Delocalized Plastic Flow in Proton-Irradiated Monolithic Metallic Glasses. *Sci. Rep.* **6**, 23244; doi: 10.1038/srep23244 (2016).

## Supplementary Material

Supplementary Movie S1

Supplementary Movie S2

Supplementary Movie S3

Supplementary Movie S4

Supplementary Information

## Figures and Tables

**Figure 2:**
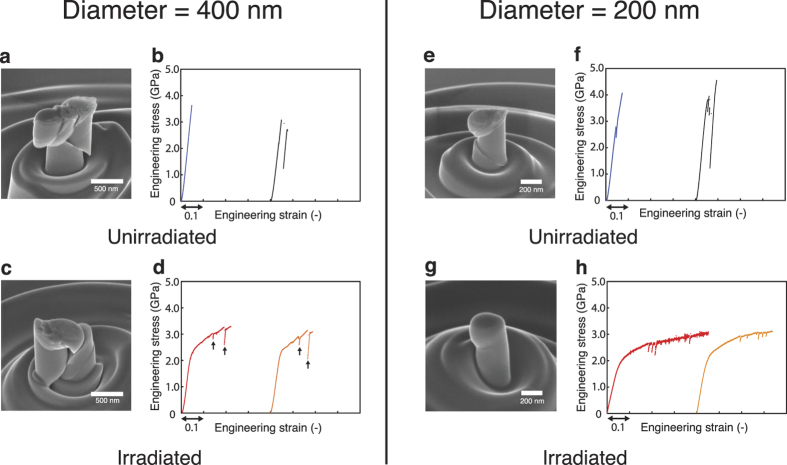
Nano-compression results for proton-irradiated and unirradiated metallic glass specimens. (**a**,**c**) Representative SEM images for unirradiated (**a**) and proton-irradiaated (**c**) metallic glass specimens with 400 nm diameter. (**b**,**d**) Engineering stress and strain curves of nano-compression experiments on 400 nm specimens. (**e**,**g**) Representative SEM images for unirradiated (**e**) and proton-irradiaated (**g**) metallic glass specimens with 200 nm diameter. (**f**,**h)** Engineering stress and strain curves of nano-compression experiments on 200 nm specimens.

**Figure 4:**
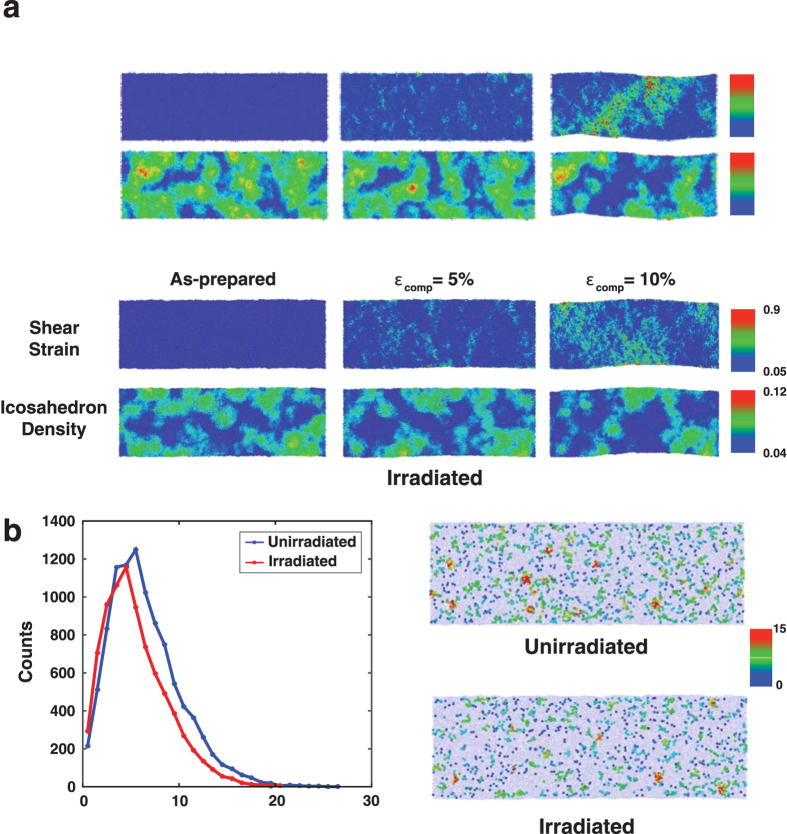
Interpenetrating icosahedral clusters and their influence on the mechanical behavior analyzed by MD simulation for 10 nm diameter metallic glass sample. (**a**) Correlation between the shear strain and icosahedron density as a function of compressive strain. (**b**) The histogram showing the number of icosahedra belonging to an icosahedral cluster of a given size. (**c**) Images showing icosahedral cluster size distribution. Atoms belonging to non-icosahedral short range orders are excluded from the images.
