# Supplementary material for: Delocalized Plastic Flow in Proton-Irradiated Monolithic Metallic Glasses
Source: Sci Rep. 2016 Mar 18;6:23244. doi: 10.1038/srep23244 (PMC4796856; doi:10.1038/srep23244)
Supplement: Supplementary Information [file srep23244-s5.pdf]

SUPPLEMENTARY INFORMATION

Delocalized Plastic Flow in Proton-Irradiated Monolithic Metallic Glasses

Jaewon Heo,[1,*] Sunghwan Kim,[2,*] Seunghwa Ryu,[2,†] and Dongchan Jang[1,†]

[1]*Department of Nuclear and Quantum Engineering*
Korea Advanced Institute of Science and Technology
Daejeon, South Korea 34141

[2]*Department of Mechanical Engineering*
Korea Advanced Institute of Science and Technology
Daejeon, South Korea 34141

This file includes:

Supplementary Movies' Captions S1-S4;

Supplementary Figures S1-S2;

* These authors contributed equally to the work.

† Correspondence and requests for materials should be addressed to S.R. (ryush@kaist.ac.kr) and D.J. (dongchan.jang@kaist.ac.kr)

SUPPLEMENTARY MOVIES' CAPTIONS

Supplementary Movie S1. MD movie showing strain evolution for unirradiated metallic glass sample.

Supplementary Movie S2. MD movie showing strain evolution for irradiated metallic glass sample.

Supplementary Movie S3. MD movie showing icosahedral density evolution for unirradiated metallic glass sample.

Supplementary Movie S4. MD movie showing icosahedral density evolution for irradiated metallic glass sample.

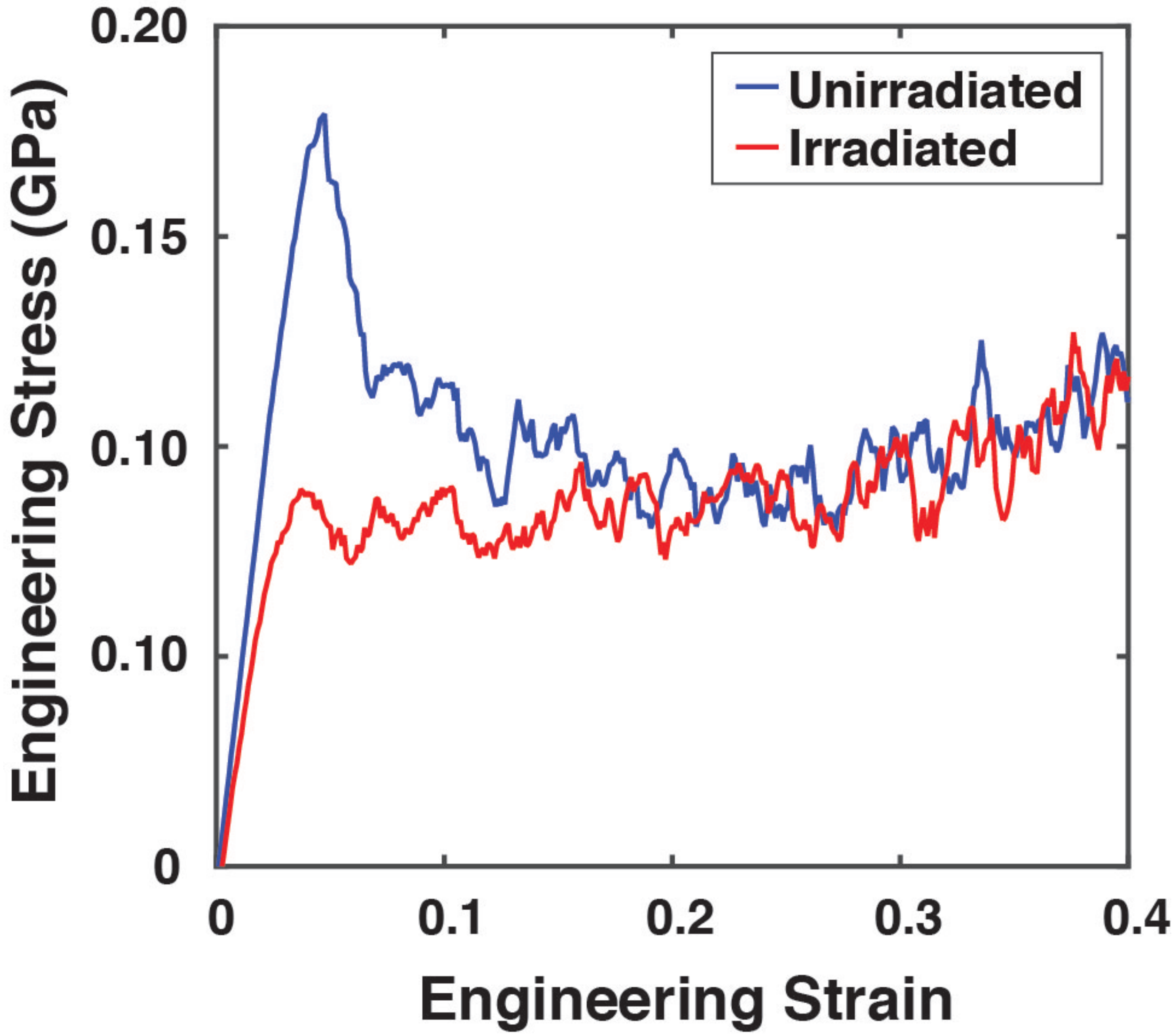

Supplementary Figure S1. **Atomistic modeling structural change of the specimen under irradiation and compressive loading for 5 nm diameter metallic glass sample.** Compressive stress-strain curves before and after irradiation. Inset represents the atomic shear strain distribution after 10% strain.

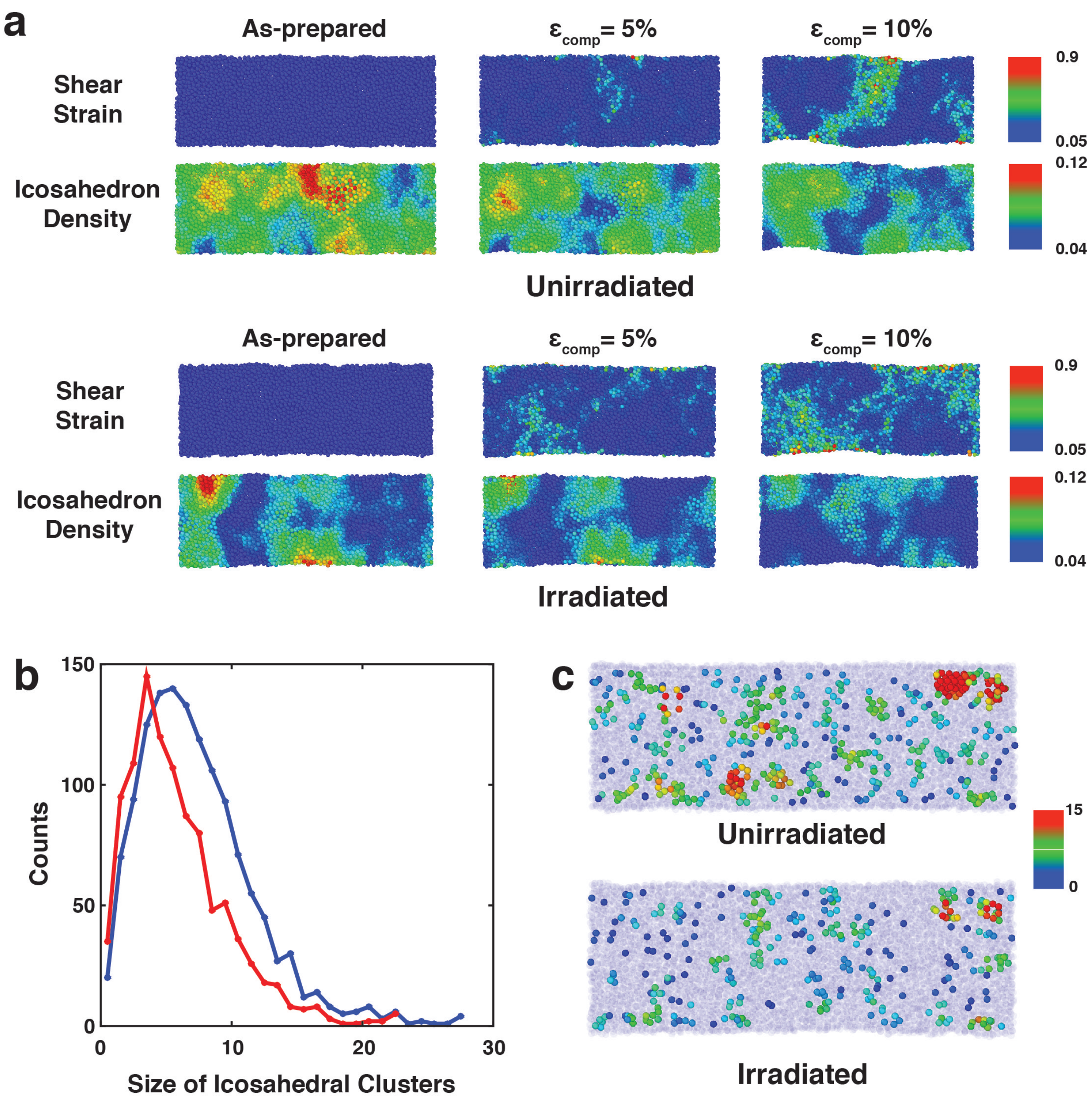

Supplementary Figure S2. **Interpenetrating icosahedral clusters and their influence on the mechanical behavior analyzed by MD simulation for 5 nm diameter metallic glass sample.** **a,** Correlation between the shear strain and icosahedron density as a function of compressive strain. **b,** The histogram showing the number of icosahedra belonging to an icosahedral cluster of a given size. **c,** Images showing icosahedral cluster size distribution. Atoms belonging to non-icosahedral short range orders are excluded from the images.
